# AI-Generated Antibiotic Therapies for Acute Periprosthetic Joint Infections with Implant Retention in Comparison with an Interdisciplinary Team

**DOI:** 10.3390/antibiotics15010025

**Published:** 2025-12-29

**Authors:** Alberto Alfieri Zellner, Tamaradoubra Tippa Tuburu, Alexander Franz, Jonas Roos, Frank Sebastian Fröschen, Gunnar Thorben Rembert Hischebeth

**Affiliations:** 1 Department of Orthopedics and Trauma Surgery, University Hospital Bonn, 53127 Bonn, Germanyalexander.franz@ukbonn.de (A.F.); jonas.roos@ukbonn.de (J.R.); 2 Department of Trauma and Orthopedic Surgery, BG Klinik Ludwigshafen, 67071 Ludwigshafen, Germany; 3 Institute of Medical Microbiology, Immunology and Parasitology, University Hospital Bonn, 53127 Bonn, Germany; hischebeth@microbiology-bonn.de

**Keywords:** LLM, AI, antibiotic therapy, PJI, interdisciplinary care, arthroplasty, hip, knee

## Abstract

**Background:** Periprosthetic joint infections (PJI) represent a serious complication following joint arthroplasty and require, in addition to surgical intervention, a targeted antibiotic therapy. The aim of this study was to compare microbiological recommendations for the antibiotic treatment of fictitious PJI patients generated by an artificial intelligence (AI) system with those of an interdisciplinary team (IT) consisting of microbiologists and orthopedic surgeons. The differences between the recommendations suggested by AI and the IT were analyzed with regard to the suggested agents and duration of antibiotic therapy. **Methods:** Based on meta-analyses, a cohort of 100 fictitious patients with acute early- and acute late-onset PJI was created, reflecting the typical demographic data, comorbidities and pathogen profiles of such a population. This information was input into the AI system ChatGPT (OpenAI, GPT-5 “Thinking mode” accessed via ChatGPT Plus, San Francisco, CA, USA) to generate corresponding recommendations. The objective was to use these profiles to obtain recommendations for definitive antibiotic therapy, including daily dosage, intravenous and oral treatment durations. Simultaneously, the same fictitious patient data were reviewed by the IT to produce their own recommendations. **Results:** The results revealed both concordances and discrepancies in the selection of antibiotics. Notably, in cases involving multidrug-resistant organisms and more complex clinical scenarios, the AI-generated recommendations were incongruent with those of the IT, with estimated percentage agreement ranging from 0–33%. In straightforward clinical scenarios with monomicrobial infections, AI reached an estimated percentage agreement of up to 57% (95%-CI [0.47–0.67]). Furthermore, AI consistently recommended 12 weeks of therapy duration vs. six weeks usually recommended by the IT. **Conclusions:** The study provides important insights into the potential and limitations of AI-assisted decision-making models in orthopedic infection treatments. Consultation of AI is universally accessible at all times of day, which may offer a significant advantage in the future for the treatment of PJI. This kind of application will be of particular interest for institutions without in-house microbiology services. However, from our perspective, the current level of incongruence between the AI-generated recommendations and those of an experienced interdisciplinary team remains too high for this approach to be clinically implemented at this time. Furthermore, AI lacks transparency regarding the sources it uses to inform about its decision-making and therapeutic recommendations, currently carries no legal weight and clinical implementation is severely hindered by restrictive privacy laws regarding health care data.

## 1. Introduction

Periprosthetic joint infection (PJI) remains one of the most devastating complications following joint arthroplasty. Despite advances in surgical techniques, sterilization protocols, and perioperative care, the incidence of PJI continues to present significant challenges to orthopedic and infectious disease teams worldwide. The impact of PJI is substantial from a clinical standpoint and in terms of patient outcomes and healthcare system burdens [1,2]. Patients with PJI often face prolonged hospital stays, repeated surgical interventions, and, in many cases, implant failure [3]. Moreover, PJI is associated with increased morbidity and mortality. Zmistowski et al. could show that the five-year mortality rate after PJI may be comparable to or even exceed that of several common cancers, including melanoma and breast cancer [4].

The management of PJI varies depending on the timing of infection onset, the stability of the prosthesis, and the causative organism. In acute postoperative or acute hematogenous infections, a treatment strategy involving debridement, antibiotics, irrigation and implant retention (DAIR) is frequently employed [5]. When applied appropriately, DAIR offers the potential to eradicate infection while preserving the prosthesis, thus reducing the need for more invasive revision procedures. However, the success of this approach is highly dependent on accurate pathogen identification, timely surgical intervention, and the selection of effective antimicrobial therapy [6].

Therefore, antibiotic treatment represents a cornerstone of PJI management, complementing surgical strategies. Empiric therapy should be initiated promptly, yet definitive antibiotic regimens should ideally be tailored based on microbiological culture results and antibiotic susceptibility profiles. Given the complexity of these cases, especially when dealing with multidrug-resistant organisms or polymicrobial infections, the selection of antibiotics often requires input from a multidisciplinary team [7]. This typically includes infectious disease specialists, orthopedic surgeons, clinical microbiologists, and pharmacists. Such collaborative decision-making is critical in optimizing patient outcomes and minimizing adverse effects associated with long-term antibiotic use [5]. Interdisciplinary decision making in antibiotic therapy is considered valuable and should be performed in complex interdisciplinary cases [8]. This interdisciplinary approach, which is a valuable addition to the patient’s treatment, is not always available and/or time consuming for the relevant disciplines.

In recent years, artificial intelligence (AI) has emerged as a promising tool in the medical field, with applications ranging from diagnostic imaging to treatment optimization [9,10]. Computer systems that can perform tasks that would usually require human intelligence (such as reasoning and learning) are referred to as artificial intelligence. A large language model (LLM) is a type of AI trained on large amounts of text to understand and generate human-like language. In the field of diagnostics for PJI, AI has been able to achieve a diagnostic accuracy with 99.3% sensitivity and 99.5% specificity based on 10 synovial fluid biomarkers analyzed [11]. Machine learning algorithms have demonstrated to work as a prognostic tool for surgical site infections in total knee arthroplasty, with an area under the receiver operating characteristic curve = 0.84 [12].

Artificial intelligence as a tool for therapy optimization has been explored in the field of infectious disease: a review performed by Hudu et al. (2025) has argued that implementing AI may improve the development of patient specific treatment strategies [13]. This could therefore be applied to the personalized treatment strategies needed for antibiotic therapy in complex patients with periprosthetic joint infections. For orthopedic surgeons, the use of AI-driven platforms to support antibiotic decision-making in complex infections such as PJI should be a huge topic of interest. In centers where microbiological expertise is limited or unavailable, AI-generated antibiotic recommendations could offer a valuable supplement to standard care by leveraging large datasets and predictive models to suggest personalized and effective, as well as safe treatment options. These technologies may assist in narrowing the gap in care quality between high-resource and low-resource hospitals, thereby contributing to more standardized and evidence-based approaches to PJI management and reducing the need for interdisciplinary meetings and case discussions, which can be time intensive and costly [14].

This study explores a freely available LLM in generating personalized antibiotic therapies in the context of acute PJI of the hip and knee with respect to the most common comorbidities in these patients. We explored the suggested therapies for intravenous and oral therapy courses, as well as biofilm-specific antibiotic therapy recommendations with rifampicin.

## 2. Results

### 2.1. Cohort Demographics and Microbiological Profiles

The synthetically generated cohort consisted of n = 52 female and n = 48 male patients. The infected joints were n = 43 hips and n = 57 knees. On average, the patients were 67.58 (±9.13) years old and had the comorbidities shown in Table 1.

The microbiological profiles of the cohort were as follows: n = 76 (76%) patients had a monomicrobial infection, n = 24 (24%) patients had a polymicrobial infection with two or more pathogens. The causative pathogens were Gram-positive in 77% (n = 100) of the cases, whilst Gram-negative bacteria made up 22% (n = 28) of the pathogens. Lastly, fungi were also present in the cohort with a prevalence of 1.54% (n = 2). In the cohort of 100 patients, there were a total of 130 pathogens, due to the polymicrobial infections present in n = 24 patients (n = 18 patients with n = 2 pathogens, n = 6 patients with n = 3 pathogens). The most common pathogens were coagulase-negative *Staphylococci* and *Staphylococcus aureus* with n = 36 (27.69%) each. The *Staphylococcus aureus* group consisted of n = 26 methicillin-susceptible *Staphylococcus aureus* (MSSA) and n = 10 methicillin-resistant *Staphylococcus aureus* (MRSA). Detailed information on the pathogens assigned to the fictitious cohort is presented in Table 2.

Within the 100 patients, there were a total of n = 19 patients with at least one difficult-to-treat pathogen, according to the Proimplant definition [15].

### 2.2. Antibiotic Treatment Protocols

For the intravenous course of antibiotic therapy, which was consistently recommended for a duration of two weeks by AI and the interdisciplinary team (except in cases where no oral antibiotic was available), AI most frequently recommended the use of vancomycin in a total of n = 46 patients. The most frequently suggested antibiotic used by the IT was also vancomycin in a total of n = 30 patients. ChatGPT 5 suggested a total of n = 18 antibiotic agents which was more than the IT, which suggested a total of n = 9 antibiotic agents. The specific agents and their respective quantities suggested are demonstrated in Table 3.

The estimated percentage agreement per clinical case requiring one intravenous antibiotic agent was 50% (95%-CI [0.4–0.6]). It dropped to 33% (95%-CI [0.06–0.6]) for patients requiring two antibiotic agents and was, finally, 0% in clinical cases that required three antibiotic agents. The Cohen’s kappa coefficient was 0.360 for cases with one antibiotic agent, 0.200 for cases with two antibiotic agents and could not be detected due to 0% estimated percentage agreement in complex cases requiring three antibiotics.

For the oral course of antibiotic therapy, which was consistently recommended for a duration of four weeks by the IT and ten weeks by AI (except in cases where no oral antibiotic was available), AI most frequently recommended the use of linezolid in a total of n = 36 patients. The most frequently suggested antibiotic used by the IT was levofloxacin in a total of n = 48 patients. The LLM suggested a total of n = seven antibiotic agents, the same number that the IT suggested (n = seven antibiotic agents). The specific agents and their respective quantities suggested are demonstrated in Table 4.

The estimated percentage agreement per clinical case requiring one oral antibiotic agent was 57% (95%-CI [0.47–0.67]). It dropped to 29% (95%-CI [0.05–0.54]) for patients requiring two antibiotic agents and was, finally, 0% in clinical cases that required three antibiotic agents. The Cohen’s kappa coefficient was 0.476 for cases with one antibiotic agent, 0.516 for cases with two antibiotic agents and could not be detected due to 0% estimated percentage agreement in complex cases requiring three antibiotics.

The antibiofilm antibiotic therapy with rifampicin was investigated separately from the above-mentioned data. The IT recommended rifampicin use in n = 46 clinical cases, whereas the AI recommended its use in n = 37 cases. Here, the estimated percentage agreement per clinical case was 75% (95%-KI [0.66–0.84]) with a Cohen’s kappa coefficient of 0.495. This category marked the best results in terms of AI and IT congruency.

## 3. Discussion

The successful treatment of PJI relies on, next to surgical treatment, appropriate antibiotic therapy. Optimal treatment decisions typically rely on interdisciplinary case discussions involving orthopedic surgeons and infectious-disease specialists/clinical microbiologists. Such expert boards are not uniformly or promptly available across all hospitals and regions, and access is often limited outside regular working hours [16]. This structural gap creates variability in the quality of antibiotic decision-making, particularly in smaller hospitals where such interdisciplinary teams are not established for routine clinical decision-making. Large language models may offer a potential solution to this problem, which is why this topic of research is so relevant. Scientific evidence that LLM-generated antibiotic treatment suggestions for acute PJI are equal to those of an established interdisciplinary team is still lacking. A study by Borgonovo et al. (2025) looked into solving complex clinical cases of osteoarticular infections, where results vary but achieved up to 94.4% accuracy [17]. Another study by Draschl et al. (2023) found that ChatGPT 5 can give valuable insights into the treatment suggestions for PJI, but that expert clinicians are needed to rate the information provided in order not to fall for misleading outputs with factual errors in complex cases [18].

### 3.1. Microbiological Profiles

The distribution of monomicrobial and polymicrobial infections observed in this study respects the patterns reported in the existing literature. Similarly, the relative frequencies of individual pathogens align well with previously published epidemiologic profiles of periprosthetic joint infection (PJI) [19,20]. Consistent with most studies focusing on acute PJI, *Staphylococcus aureus* and CNS were responsible for the most cases in our cohort. These similarities to the real microbiological trends in PJI research allow the maximization of the value of the findings from our fictional cohort.

Culture-negative PJI continues to present a significant clinical challenge, particularly because its diagnosis and management rely heavily on empiric treatment strategies. This study design, where each case needed a pathogen with an accompanying susceptibility profile, was not suited to investigate culture-negative cases. In the literature, culture-negative PJI makes up 4.7–32.5% of all PJI cases, which reduces the validity of these findings [19,21,22]. In routine clinical practice, culture-negative PJI is managed with broad empiric antibiotic regimens. The selection of these empiric antibiotic agents depends entirely on institution-specific microbiological profiles and susceptibility patterns, as well as the individual profile of the patient that is treated for a culture-negative PJI. As diagnosis-related (PJI-related) susceptibility profile data are protected health information and vary considerably between centers, a large language model cannot possibly make context-appropriate therapeutic recommendations in these cases. A future solution to this problem could be running a local, hospital-based LLM with access to patient, pathogen and susceptibility data.

### 3.2. Therapy Duration and Antibiotic Agents

A notable critique of the current AI-based recommendations in this study is the lack of transparency regarding the information and internal rationale guiding its therapeutic decision-making. This is reflected in the—from a clinical point of view—inconsistent antimicrobial recommendations produced within short time intervals (whilst feeding the LLM several cases in a row, for example). Here, despite near identical clinical inputs, the LLM suggested cefazolin in some scenarios and flucloxacillin in others for patients with acute PJI due to MSSA and no relevant comorbidities or allergies. This happened without the LLM explaining its reasoning for divergent choices in, clinically, the same cases. Similarly concerning were instances in which vancomycin was recommended for patients with chronic renal insufficiency, a practice that, in a clinical setting, carries substantial risk of further acute kidney injury due to its nephrotoxicity and would be considered unsafe in vulnerable patient populations. Nevertheless, the LLM did correctly adjust the dosing for this recommendation.

In other cases, the LLM issued recommendations for antibiotic agents that were not represented in the available susceptibility profiles of the pathogens provided to the LLM. Following these recommendations without further susceptibility testing may result in inappropriate antibiotic therapy and may have promoted resistance development by some pathogens. Our IT, on the other hand, would demand testing of further antibiotic agents, when necessary. The LLM skipped this part. Moreover, certain regimens were excessively broad, including suggestions such as colistin or meropenem when narrower agents like ampicillin/sulbactam would have been clinically appropriate. Another interesting finding is that the LLM preferred cephalosporins (especially Ceftriaxone) for Gram-negative infections, whereas the IT largely relied on penicillin derivatives.

The three most frequently suggested antibiotic agents for the oral phase were levofloxacin, linezolid, and amoxicillin. Notably, with respect to linezolid, the algorithm did not restrict total treatment duration (intravenous plus oral application of linezolid) to the commonly accepted 28-day maximum specified in the prescribing information, thereby formally exceeding the approved labeling for prolonged use [23]. According to current surveillance data from the Robert Koch Institute, no linezolid resistance in *S. aureus* has been reported over the past three years, whereas levofloxacin resistance in *S. aureus* has shown a favorable declining trend from 11.7% to 10.2% over the same period (2022–2024) [24]. Within the CNS, the main representative *Staphylococcus epidermidis* likewise demonstrated a decrease in levofloxacin resistance from 45.3% to 42.9%, while resistance to linezolid remained consistently low at 1.0–1.2% in the three-year time window from 2022–2024 [25]. These epidemiological data of the resistance patterns of these antibiotic agents support their continued clinical utility for oral step-down therapy in acute PJI, provided that linezolid is used within approved duration limits and under appropriate drug monitoring.

During this study, an antibiotic therapy for a duration of six weeks in total was established by the IT as the standard protocol. This reflects the clinical practice, which has used this therapy duration as part of DAIR treatment for years now [25]. This decision is based on results in the literature, where the non-inferiority of six weeks of antibiotic treatment was shown compared to 12 weeks of antibiotic therapy. Chaussade et al. (2017) published their results and found no significant differences between six weeks and twelve weeks of antibiotic therapy [26]. A review published in The Journal of Bone and Joint Surgery by Miller et al. (2020) found that there is no disadvantage to a 6-week course of antibiotic therapy after DAIR [27]. The AI consistently opted for a 12-week course of antibiotic therapy, which was one of the main differences between the treatment suggestions of the IT and AI. Upon specific request to explain the therapy duration of 12 weeks, the LLM based its decision on the 2013 ISDA guidelines for PJI treatment [6]. Furthermore, the LLM cited the original article by Bernard et al. (2021), which was published in The New England Journal of Medicine, a position paper from Sigmund et al. (2025) (in which the 12-week therapy duration is based on the findings from Bernard et al.) and a narrative review from Lora-Tamayo et al. (2024) [28,29,30].

### 3.3. Rifampicin in Acute PJI

In the context of acute PJI managed with DAIR in the presence of *Staphylococcus* spp., rifampicin is regarded as a cornerstone of biofilm-active therapy [31,32]. Within this framework, the AI system generally recommended initiating rifampicin only once the surgical wound was clinically healed and free of drainage. This conservative timing is consistent with current evidence and with the practice of the IT and minimizes the risk of selecting rifampicin-resistant staphylococcal strains [32,33].

Among all therapeutic decision categories evaluated, the use of rifampicin showed the highest percentage agreement between the AI and the IT, accompanied by a moderate Cohen’s kappa coefficient of 0.495. This combination suggests a relatively high level of concordance. The decision to start rifampicin in a clinical case, though, is simple: the choice is essentially dichotomous (rifampicin yes/no) and then dependent on the susceptibility profile of *Staphylococcus* isolates. Therefore, the remaining 25% of cases with discordant rifampicin recommendations between AI and IT require careful interpretation. Arguably, this degree of non-concordance is more concerning than the lower percentage agreement observed for oral and intravenous companion agents. For oral and intravenous antibiotic choices, equivalent or near-equivalent options frequently exist, and divergent recommendations (e.g., choice A vs. choice B) do not necessarily imply that one regimen would have been clinically ineffective. In contrast, disagreement in the rifampicin domain means that the therapy was effectively right or wrong. From a clinical perspective, a 25% discrepancy in rifampicin decisions, therefore, may carry greater weight than a similar or even larger discrepancy in the choice among several acceptable systemic agents. In summary, patients with acute PJI need, whenever possible, a targeted anti-biofilm therapy to maximize treatment success [34]. This concludes that basing the treatment on recommendations generated by an LLM is not a viable option at this stage.

### 3.4. Limitations

Despite the decision support in orthopedic infection management that an LLM could give, several substantial limitations currently limit its routine clinical use, particularly within the German healthcare context. Firstly, the applications of the findings from this study are severely limited by legal and data protection constraints. Under current interpretations of German privacy laws, patient-level clinical data cannot be processed by non-certified AI systems for the purpose of medical decision making, especially if data leave the secure institutional environment or are handled by third-party providers with data storage at unknown locations. As a result, real-world integration of AI algorithms into clinical workflows is significantly impacted. The review articles from Maritati et al. (2025) and Vulpe et al. (2025) touch on this topic in regard to the diagnostic and prognostic capabilities of AI [9,10]. Here, emphasis is laid on the value of the great amount of patient data available per clinical case, with the limited access that is granted to an AI due to restrictive privacy laws, which can significantly hinder AI effectiveness in a clinical setting [9,10]. Secondly, the present work is based on a fictional patient cohort. While synthetic or simulated data can be used for a technical demonstration of AI models regarding the antibiotic therapy suggestion in patients, they cannot fully reproduce the complexity and, especially, the variability, with dynamic clinical courses in real clinical populations. For this reason, this study design cannot simulate clinically relevant scenarios such as intolerance, allergic reactions, or significant side effects. Finally, it should be emphasized that AI-generated therapeutic suggestions currently carry no legal weight. If a clinician follows an AI-based suggestion and an error occurs, the AI system would not be an accountable decision-making entity, transferring the full responsibility to the treating physician. Furthermore, Chat-GPT-5, as the used LLM, is not medically certified for the presented use.

While using Cohen’s Kappa agreement for the comparison of the antibiotic regimens suggested by AI and the IT, this method is subject to the Cohen’s Kappa paradox, which can affect results. This paradox refers to situations in which observed agreement is high, yet Kappa is low. It occurs because Kappa corrects for agreement expected by chance, and the chance agreement term can become very large under certain data conditions, so that the result of the term can be lowered under these circumstances [35].

### 3.5. Cohort Formation and Dataset

One limitation of the study is its reliance on a cohort of fictional patients, a choice necessitated by strict German data-protection regulations that require individual informed consent for the use of real patient information in addition to specific knowledge of where patient data are stored, with storage only permitted on German soil and how to delete them fully. Although this approach restricts direct “real-world” use at this stage, the constructed cohort demonstrates strong comparability to comparable studies with respect to age distribution, comorbidity profiles, and distribution of affected joints [25,36]. This is due to the fact that cohort generation was based on a meta-analysis by Choong et al. (2022), ensuring that key epidemiologic and clinical parameters reflect those of previously published patient populations [37]. A notable strength of the study is the comparison to the antibiotic recommendations of an interdisciplinary team from a referral center for PJI treatment.

## 4. Materials and Methods

### 4.1. Study Design and Objective

This simulation-based study aimed to evaluate the concordance between large language model (LLM)-generated recommendations and expert clinical decision-making in the context of definitive antibiotic therapy for acute periprosthetic joint infections (PJI) treated with debridement, antibiotics, and implant retention (DAIR). Given ethical and data privacy concerns, all patient data were synthetically generated based on real-world epidemiological distributions to ensure a representative and controlled dataset without involving actual patients. For this purpose, the data presented in a meta-analysis conducted by Choong et al. (2022) was used [37].

### 4.2. Synthetic Patient Cohort Creation

A total of 100 fictitious patient profiles were created to simulate cases of acute PJI treated with DAIR. Each synthetic patient profile included the following variables:

- Age
- Relevant comorbidities (diabetes mellitus, chronic renal failure, chronic obstructive pulmonary disease (COPD), heart disease, liver disease, neurological comorbidities)
- Allergy to penicillin, fluoroquinolones
- Infectious pathogen
- Pathogen-specific antibiotic susceptibility profile

We defined the number of patients allergic to penicillin following a study from Luintel et al. (2025) with 9% [38]. We defined the number of patients allergic to fluoroquinolones following a study from Wall et al. (2018) with 2% [39].

The distributions of pathogens and comorbidities were modeled based on pooled prevalence data from a recent meta-analysis of studies focusing on acute PJIs managed with DAIR [37]. Common pathogens (e.g., *Staphylococcus aureus*, coagulase-negative *staphylococci*, *enterococci*, Gram-negative pathogens) and comorbidity profiles were included in proportions reflective of clinical practice. The susceptibility profiles were created using the data from our center (Fröschen et al., 2022) for periprosthetic joint infections [20]. Difficult-to-treat pathogens were defined as rifampicin resistant *staphyloccoci*, ciprofloxacin-resistant Gram-negative bacteria, and yeasts in line with the current Proimplant definition [15].

### 4.3. Language Model-Based Recommendation Process

Each synthetic case was separately input into one large language model (ChatGPT, OpenAI, GPT-5 model in thinking mode, accessed via ChatGPT Plus, San Francisco, CA, USA), in English language, in the time frame from 1 September 2025–1 October 2025: The input prompt for each case included a complete and standardized clinical summary, which was written in the same way for each input following this structure: patient age, clinical diagnosis (Acute PJI treated with DAIR), comorbidities, known drug allergies, pathogen identified, and its full susceptibility profile. The models were then asked to recommend a complete definitive antibiotic regimen, including both intravenous (IV) and oral (PO) options suitable for transition therapy.

Prompts were standardized and manually reviewed to ensure consistency. The models were asked to provide:

- Antibiotic agents
- Route and duration of administrations (IV and PO)

An exemplary prompt for a monomicrobial acute PJI is listed below:

“Please give a case summarization and antibiotic therapy suggestions (including antibiotic agent, dosage, therapy duration, antibiofilm treatment) for the following patient profile with an acute periprosthetic joint infection that is set to be treated with DAIR (Debridement, antibiotics and implant retention):

Age45 years oldJointHipGenderMaleComorbiditiesCOPD, Heart diseaseAllergiesnonePathogenMSSA, results of antibiotic susceptibility testing listed in the following table”

### 4.4. Expert Panel Comparison

In parallel, the same 100 fictitious patient cases were presented to a clinical expert team comprising:

- Two orthopedic surgeons with subspecialty training in septic revision arthroplasty
- Two clinical microbiologists with expertise in musculoskeletal infections (interdisciplinary team, IT)

All experts worked at a tertiary referral center specializing in septic revision arthroplasty and routinely participated in infection management rounds. The experts were blinded to the LLM-generated suggestions and asked to independently propose a definitive antibiotic therapy plan for each case, following international treatment guidelines and institutional protocols.

### 4.5. Sample Size Justification

This study was designed as a concordance study with the primary objective of estimating agreement (percentage agreement and Cohen’s κ) between the LLM and the expert consensus of the IT. We predefined n = 100 synthetic cases as a feasibility-based sample size: for percentage agreement, n = 100 yields a 95% confidence interval half-width of approximately ±0.10 at *p* = 0.50 (and narrower for higher agreement), and it allows estimation of Kappa with acceptable uncertainty for an exploratory assessment. In addition, n = 100 ensured inclusion of infrequent but safety-relevant clinical scenarios (e.g., antibiotic allergies and difficult-to-treat pathogens) while maintaining a feasible expert review workload. Furthermore, the sample size of n = 100 is in line with study cohorts of acute PJI treated with DAIR [25,40]. Sample-size planning methods for κ based on interval estimation/precision have been described in the reliability literature and were used to inform this design choice.

### 4.6. Outcome Measures

The primary outcome was the concordance between LLM-generated antibiotic recommendations and expert consensus. Concordance was measured as percentage agreement and Cohen’s kappa coefficient.

Secondary analyses included:

- Frequency of recommendations consistent with guideline-based therapy
- Instances of potentially inappropriate recommendations of AI (e.g., due to allergies, resistance, or known contraindications)

### 4.7. Data Management and Ethics

All data used in this study were synthetically generated and did not involve real patients, thereby eliminating the need for institutional review board approval or informed consent. The study design adhered to ethical standards for simulation and modeling studies.

## 5. Conclusions

In conclusion, our findings suggest that ChatGPT 5 may serve as a supportive tool for antibiotic therapy in non-complex, monomicrobial PJIs when immediate access to an interdisciplinary consultation with an expert clinical microbiologist and/or orthopedic surgeon specialized in revision arthroplasty is limited. In such scenarios, LLM-based recommendations can bridge the gap until formal consultation with orthopedic surgeons and clinical microbiologists becomes available. It is important to acknowledge that these recommendations are not a substitute for a case-based, individual antibiotic therapy discussed by an interdisciplinary panel of experts. In contrast, in complex clinical cases and/or polymicrobial infections, as well as in patients with relevant allergies and/or significant comorbidities, AI-generated antibiotic regimens were frequently inconsistent and clinically incoherent compared with those proposed by the interdisciplinary team that evaluated the cases in this study. This discrepancy has particular consequences in the selection of biofilm-active antibiotic regimens, where an incongruence rate of 25% was observed. This margin of incongruence is unacceptable in the context of implant-associated infections. Beyond these performance limitations, stringent healthcare data privacy laws and regulations considerably restrict the “real-world” clinical deployment of LLMs. Persistent concerns regarding secondary data use, incomplete data deletion and loss of patient control over personal health information are factors that, at present, LLMs can only be recommended as an additional decision-support tool for individual patients.

## Figures and Tables

**Table 1:** Demographic data of the n = 100 patients with acute periprosthetic hip and knee infections. SD = standard deviation; COPD = chronic obstructive pulmonary disease.

Demographic Data	Hip	Knee	Total
Total Number of Patients	43	57	100
male	23	25	48
female	20	32	52
Age, mean ± SD, (Range)	66.81 (±9.53)	68.16 (±8.85)	67.58 (±9.13)
Renal insufficiency	3	5	8
Diabetes	9	12	21
Neurological diseases	6	10	16
Liver disease	1	2	3
Heart disease	10	9	19
COPD	5	6	11
Penicillin allergy	5	4	9
Fluoroquinolone allergy	1	1	2

**Table 2:** Microbiological profiles of the n = 100 patients with acute periprosthetic hip and knee infections. A total of n = 130 pathogens could be detected.

Pathogen	n	Percentage %
Coagulase negative *Staphyloccoci*	36	27.69
*Staphylococcus aureus*	36	27.69
*Streptococci*	20	15.38
*Escherichia coli*	9	6.92
*Proteus* spp.	7	5.38
*Enterobacter* spp.	7	5.38
*Enterococcus* spp.	6	4.62
*Pseuodomonas aeruginosa*	5	3.86
*Cutibacterium acnes*	2	1.54
*Candida albicans*	2	1.54

**Table 3:** The total quantity of the antibiotic agents recommended by the LLM and IT for the i.v. course of antibiotic therapy. LLM: large language model; IT: interdisciplinary team.

Agent	LLM Quantity	IT Quantity
Vancomycin	48	32
Flucloxacillin	10	2
Cefazolin	10	21
Penicillin G	9	17
Ampicillin/Sulbactam	1	12
Ceftriaxon	13	0
Meropenem	8	6
Piperacillin/Tazobactam	3	6
Daptomycin	2	5
Ampicillin	4	0
Cefepim	4	0
Gentamicin	3	0
Caspofungin	2	2
Linezolid	2	0
Colistin	1	0
Ciprofloxacin	1	0
Ceftazidime	1	0
Aztreonam	1	0
Total	123	103

**Table 4:** The total quantity of the antibiotic agents recommended by the LLM and IT for the oral course of antibiotic therapy. LLM: large language model; IT: interdisciplinary team.

Agent	LLM Quantity	IT Quantity
Levofloxacin	21	48
Linezolid	39	14
Amoxicillin	18	22
Ciprofloxacin	16	17
Cotrimoxazol	8	13
Clindamycin	4	1
Fluconazol	2	2
Total	108	117

## Data Availability

The original contributions presented in this study are included in the article; further inquiries can be directed to the corresponding author.
